# Genome-wide analysis of proline-rich extension-like receptor protein kinase (PERK) in *Brassica rapa* and its association with the pollen development

**DOI:** 10.1186/s12864-020-06802-9

**Published:** 2020-06-15

**Authors:** Guohu Chen, Jian Wang, Hao Wang, Chenggang Wang, Xiaoyan Tang, Jie Li, Lei Zhang, Jianghua Song, Jinfeng Hou, Lingyun Yuan

**Affiliations:** 1 Vegetable Genetics and Breeding Laboratory, College of Horticulture, Anhui Agricultural University, Hefei, 230036 China; 2 Anhui Provincial Engineering Laboratory of Horticultural Crop Breeding, Hefei, 230036 China; 3 Wanjiang Vegetable Industrial Technology Institute, Maanshan, 238200 China

**Keywords:** *Brassica rapa*, Comparative genome analysis, Male sterile, Proline-rich receptor-like protein kinase, Transcriptomic analysis

## Abstract

**Background:**

Proline-rich extension-like receptor protein kinases (PERKs) are an important class of receptor kinases located in the plasma membrane, most of which play a vital role in pollen development.

**Results:**

Our study identified 25 putative *PERK* genes from the whole *Brassica rapa* genome (AA). Phylogenetic analysis of PERK protein sequences from 16 Brassicaceae species divided them into four subfamilies. The biophysical properties of the *BrPERK*s were investigated. Gene duplication and synteny analyses and the calculation of *Ka*/*Ks* values suggested that all 80 orthologous/paralogous gene pairs between *B. rapa* and *A. thaliana*, *B. nigra* and *B. oleracea* have experienced strong purifying selection. RNA-Seq data and qRT-PCR analyses showed that several *BrPERK* genes were expressed in different tissues, while some *BrPERK*s exhibited high expression levels only in buds. Furthermore, comparative transcriptome analyses from six male-sterile lines of *B. rapa* indicated that 7 *BrPERK* genes were downregulated in all six male-sterile lines. Meanwhile, the interaction networks of the *BrPERK* genes were constructed and 13 *PERK* coexpressed genes were identified, most of which were downregulated in the male sterile buds.

**Conclusion:**

Combined with interaction networks, coexpression and qRT-PCR analyses, these results demonstrated that two *BrPERK* genes, Bra001723.1 and Bra037558.1 (the orthologs of *AtPERK6* (AT3G18810)), were downregulated beginning in the meiosis II period of male sterile lines and involved in anther development. Overall, this comprehensive analysis of some *BrPERK* genes elucidated their roles in male sterility.

## Background

Unique membrane receptor-like kinases (RKs) have evolved to regulate biological processes in plants [1, 2]. These plasma membrane RKs perceive various stimuli and direct them to downstream signaling networks via the phosphorylation of specific domains [3]. In *Arabidopsis*, approximately 610 RKs have been predicted, which comprise a variety of extracellular ligand-binding domains and are implicated in a wide range of functions, from plant growth and development to plant-environment interactions [2, 4, 5]. These RK proteins can be grouped into many different classes according to their extracellular domain motifs [6, 7]. For instance, the leucine-rich repeat receptor kinases (LRR-RKs) are the largest of the RK family in *Arabidopsis* and sense a diverse set of hormone signaling pathways to regulate cell proliferation and expansion, stomatal development and stem cell maintenance [5, 8–10].

Proline-rich extension-like receptor-kinases (PERKs) are a small family of RKs containing cytoplasmic kinase-like domains, transmembrane motifs and extracellular proline-rich domains [1, 11, 12]. The first *PERK* gene was identified in *Brassica napus*, which is rapidly induced by wounding, ubiquitously expressed in the stem, petals and pistils and controls root and stem branching [13, 14]. Recent work addressing the *PERK* family of *Gossypium hirsutum* has revealed the expression patterns of these genes in response to various abiotic stresses and hormonal homeostasis [15]. In addition to these functions, PERKs are thought to act as sensors/receptors to monitor changes in the cell wall during its expansion [1, 12].

In *Arabidopsis*, the *PERK* family includes 15 members, and the analysis of their expression pattern shows that several of these genes are broadly expressed, while some *AtPERK* genes are specifically expressed [16]. For example, *AtPERK1* (*NsAK*) is ubiquitously expressed, and *AtPERK2* is mainly expressed in rosette leaf veins, stems and pollen [14, 17]; *AtPERK4*, a Ca^2+^ signaling regulator, is implicated in root growth and seed germination and is highly expressed in mature pollen and pollen tubes [18–20]; *AtPERK8* and *AtPERK13* (*RHS10*) are specifically expressed in root hairs [11, 16, 21]; and *AtPERK5*, *AtPERK6, AtPERK7*, *AtPERK11* and *AtPERK12* (*IGI1*) are highly expressed in pollen but are almost undetectable in sporophytic tissues [1, 11, 22, 23].

Evidence from *Arabidopsis* shows that most *PERK*s not only contribute to cell growth and cell wall deposition but also play important roles in male-female interactions and precisely control pollen/pollen tube behaviors [1, 18]. Male gametogenesis requires several metabolic pathways, including the tapetum development, callose metabolism, anther dehiscence, pollen wall and pollen tube wall formation pathways [24–26], and defects in any of these processes can cause abnormal development and ultimately lead to male sterility [26]. However, the molecular mechanisms of *PERK*s function in pollen development, especially in male sterility, are still unknown.

Thus far, the genome-wide characterization of *PERK* genes has been reported only in cotton and *Arabidopsis* [15, 16]. Little information on the *PERK* family and their biological functional roles in *B. rapa* are available. Chinese cabbage (*B. rapa* L.) is one of the most important species in the Brassicaceae family and is widely cultivated in most parts of China [26, 27]. In this study, we performed a systematic analysis of the *PERK* family in *B. rapa*. The biophysical properties of *BrPERK* genes were determined. Furthermore, RNA-Seq data from six male-sterile plants (two male-sterile mutants [26, 28], two GMS lines [29, 30] and two *ogu*-CMS lines [27, 31]) were analyzed, and tissue-specific expression patterns were examined to identify the specific *PERK* genes related to pollen development and male sterility in *B. rapa*. The present study will contribute to a detailed understanding of the molecular and biological functions of *BrPERK* genes in male-sterile plants of *B. rapa*.

## Results

### Identification of PERK proteins

**Fig. 1:**
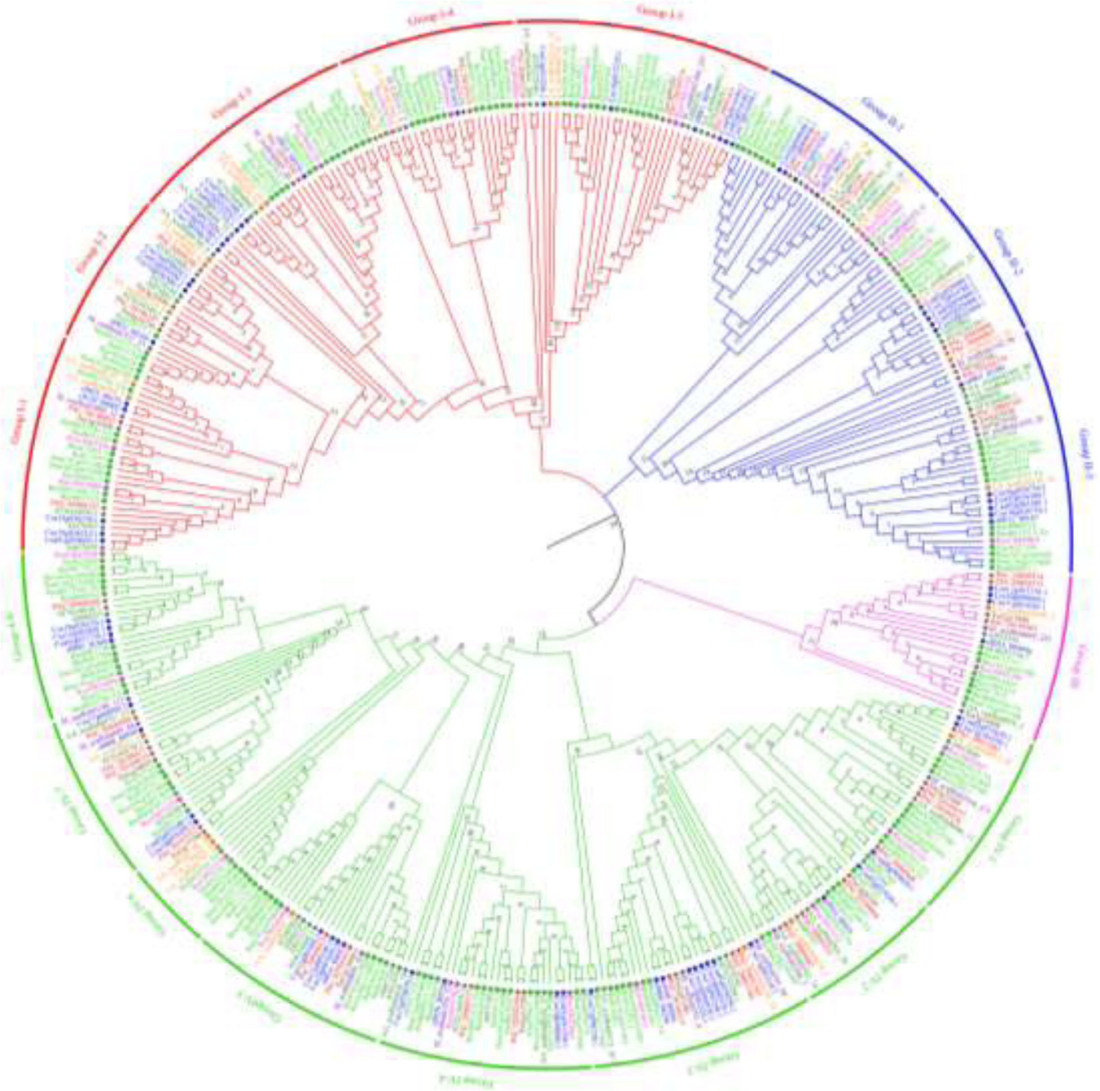
Phylogenetic analysis of PERK proteins from 16 Brassicaceae species. 418 PERK proteins are grouped into four subfamilies. The gene number of 16 Brassicaceae species in different groups was calculated in Table S2. Percent bootstrap values (1000 iterations) are indicated in every branch. The different-colored arcs indicate different groups of PERKs. The maximum likelihood (ML) tree was constructed by MEGA7 with the Bootstrap method (1000 replications) and the Jones, Taylor, and Thornton amino acid substitution (JTT) model

In our study, a total of 418 *PERK* gene family members in 16 Brassicaceae species were identified based on several confirmations. With the exception of *B. juncea* (n = 18), *B. napus* (n = 19) and *Camelina sativa* (n = 20), the *PERK* gene numbers in the other 13 species ranged from 17 to 27 (Table S1; Fig. S1), which might be related to their chromosome numbers and indicated that the *PERK* genes have undergone expansion in Brassicaceae species. A maximum likelihood (ML) tree was generated for these PERK proteins, showing that all PERKs were grouped into four subfamilies (Groups I-IV; Fig. 1, Table S2). Although Group III contained the fewest PERKs (only 22 members), it included PERK members from all Brassicaceae species, and most species exhibit only one PERK (Table S2).

**Fig. 2:**
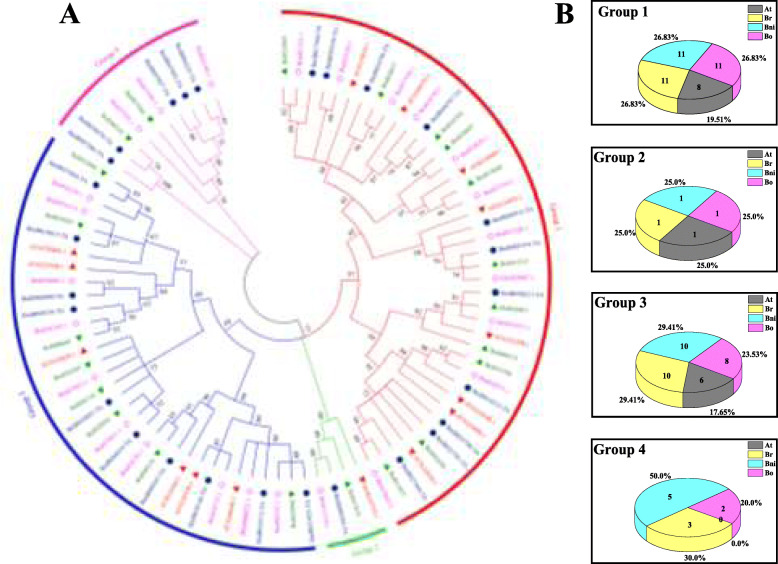
Phylogenetic analysis and distribution of PERK proteins from four plant species. **a**, Phylogenetic tree of *B. rapa*, *B. nigra*, *B. oleracea*, and *A. thaliana*. 89 PERKs from them are divided into four groups. The different-colored arcs indicate different groups of PERKs. The maximum likelihood (ML) tree was constructed by MEGA7 with the Bootstrap method (1000 replications) and the Jones, Taylor, and Thornton amino acid substitution (JTT) model. **b**, Number and percentage of PERK proteins across the four plant species within each subfamily. At, *Arabidopsis thaliana*; Br, *Brassica rapa*; Bni, *Brassica nigra*; Bo, *Brassica oleracea*

Among *Brassica* species, 25, 27 and 22 *PERK* family members were identified in *B. rapa* (AA), *B. nigra* (BB) and *B. oleracea* (CC), respectively (Table S1; Fig. S1). The phylogenetic tree of these genes along with those of *A. thaliana* showed that the total 89 PERKs could again be divided into four groups (Fig. 2a, Groups 1–4). Group 1 and Group 3 constituted the largest clades, containing a total of 65 PERKs, and accounted for 46.07 and 38.20% of the sequences, respectively. Interestingly, the PERK protein numbers of the three *Brassica* species in Groups 1–3 were almost the same (Fig. 2b), indicating that these *PERK* genes from the three *Brassica* plants may have come from a common ancestor. Furthermore, the sequence logos of the homologous domain sequences of the PERK proteins revealed that the domain sequences were highly conserved in *A. thaliana* and the three *Brassica* species (Fig. S2).

We also determined the physical and chemical characteristics of BrPERK proteins. The BrPERK proteins showed wide variation in their length, molecular weight (MW) and isoelectric point (pI) (Table S3). Moreover, the majority of the proteins were localized to the chloroplast thylakoid membrane, followed by the endoplasmic reticulum (membrane), plasma membrane, cytoplasm, nucleus and chloroplast stroma (Table S3).

### Conserved motifs, gene structures and *cis*-elements analyses

**Fig. 3:**
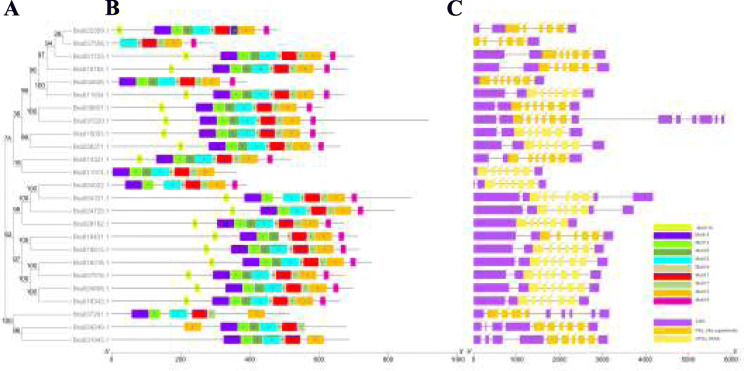
Phylogenetic relationship, gene structure and conserved motif analyses of *BrPERK* genes. **a**, Phylogenetic tree of BrPERK proteins’ sequences was generated by unrooted NJ, and the bootstrap test replicate was set as 1000 times with MEGA7. **b**, Conserved motifs of BrPERK proteins that were identified using the MEME program. Ten putative motifs in Fig. S3 are indicated by different coloured boxes. **c**, The exon-intron organization and Pkinase domain (and STKc_IRAK) of *BrPERK* genes. Black lines represent introns. Exons, Pkc_like superfamily domains and STKc_IRAK domains are represented by purple, orange and yellow boxes, respectively

The functional motifs and gene exon-intron positions were analyzed based on evolutionary tree relationships (Fig. 3). The ten most conserved motifs were identified in the BrPERK members (Fig. S3), showing that almost all of the BrPERK proteins contained the same motifs in terms of both the type and distribution patterns of the motifs, except for Bra037558 (Fig. 3b). The exon-intron organization suggested that all *BrPERK* genes showed conserved patterns of gene structure, and all conserved motifs were associated with the Pkinase domain (Pkc_like superfamily and STKc_IRAK) (Fig. 3c). Overall, the highly similar gene structures and motif distributions of the *BrPERK* members were consistent with their phylogenetic relationships (Fig. 3). *cis*-element analysis showed that several promoters contained meristem expression motifs, and a few promoters contained endosperm and seed-specific expression, cell cycle regulation and circadian control elements (Table S4; Fig. S4), indicating the roles of several *PERK* genes in plant flowering.

### Gene duplication, synteny and *Ka*/*Ks* analyses

The mapping of the *BrPERK* genes to chromosomal loci showed that an inconsistent distribution of the genes, with 1 to 5 genes being distributed on chromosomes A1-A9 (Fig. S5A; Table S3). Synteny and *Ka*/*Ks* analyses indicated that 13 paralog pairs were distributed on different chromosomes, not including the A04 and A10 chromosomes (Table S5, Fig. S5B). The *Ks* values ranged from 0.29 to 1.23, and the average duplication time of paralog pairs was indicated to be 22.84 million years ago (Mya) (Table S5, Fig. S6A), possibly because some *BrPERK* gene duplications occurred before the whole-genome triplication observed in *B. rapa*.

**Fig. 4:**
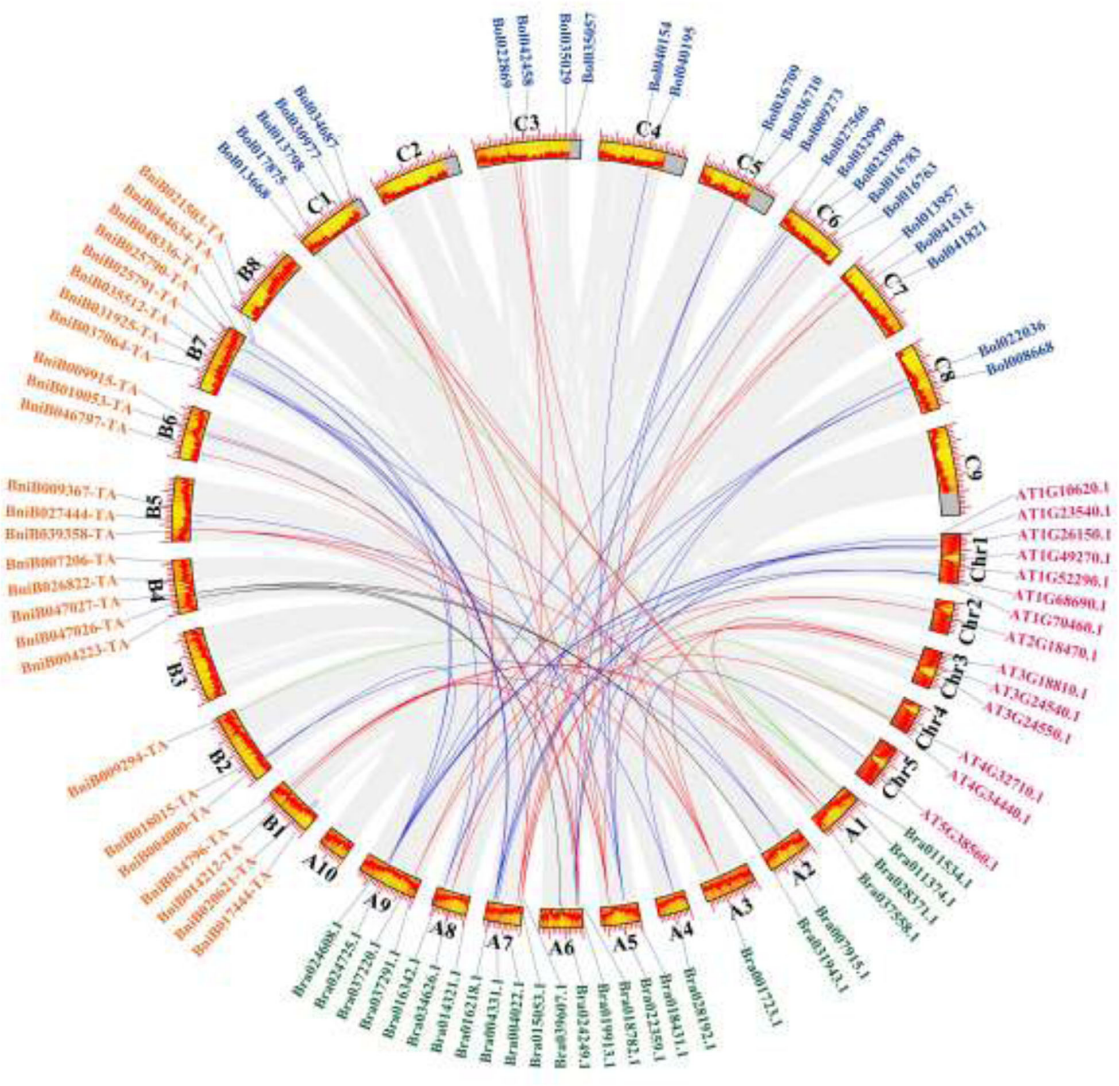
*PERK* gene duplication among *B. rapa* (AA) and *A. thaliana, B. nigra* (BB), *B. oleracea* (CC). Different color lines exhibited paralogous pairs. Chr1-Chr5 (red blocks) indicated chromosomes of *A. thaliana*; A1-A10 (green blocks), B1-B8 (orange blocks) and C1-C9 (blue blocks) represented sub-genomes of *B. rapa* (AA), *B. nigra* (BB) and *B. oleracea* (CC), respectively

Further analyses of *PERK* gene evolution and divergence among *A. thaliana* and the three *Brassica* species showed that a total of 67 orthologous gene pairs exhibited a collinear relationship (17 Br-At, 27 Br-Bni, 23 Br-Bo; Fig. 4, Table S5). These results demonstrated that the *PERK* genes of these *Brassica* species appeared to be derived from a common ancestor and that the function of these *PERK* genes of *Brassica* plants might be the same as those of *A. thaliana*. In addition, among the orthologous gene pairs, each *AtPERK* gene presented 1–3 *BrPERK* orthologous genes (Fig. 4 and S7, Table S5), suggesting that a few *BrPERK* genes had been duplicated by genome triplication. The *Ka*/*Ks* values of these gene pairs were all less than 0.35 except for one pair (Bra028371.1-Bo1013798, *Ka*/*Ks* = 0.56), and the average divergence times were estimated to be 17.12 Mya (Br-At), 11.96 Mya (Br-Bni), and 8.95 Mya (Br-Bo) (Table S5, Fig. S6B-D). These results demonstrated that the *PERK* gene pairs shared between *B. rapa* and *B. nigra*, *B. oleracea*, and *A. thaliana* had undergone strong purifying selection with limited functional divergence after whole-genome duplication (13–17 Mya [32]).

### Tissue-specific expression pattern analysis

**Fig. 5:**
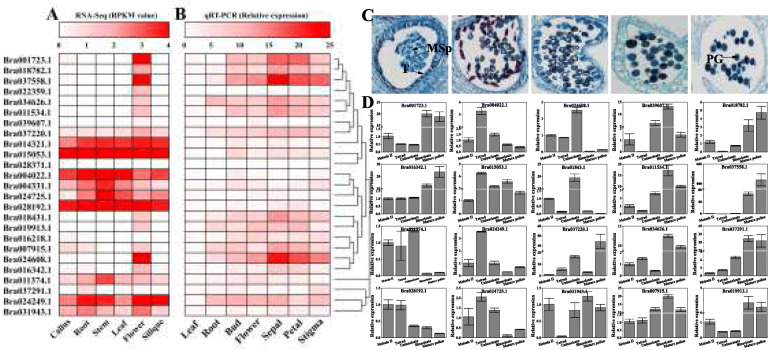
RNA-Seq and qRT-PCR analyses of the *BrPERK* genes in *B. rapa*. **a**, Illumina RNA-Seq data analysis for six tissues of *B. rapa*. **b**, qRT-PCR analysis of *BrPERK* genes in different tissues from *B. rapa*, the gene expression values of different *PERK* genes in leaf were set as 1. The bars on the top represent of RPKM values and relative expression values, respectively. **c**, Cytological observation of five stages (meiosis II period, tetrad period, uninucleate period, binucleate period and mature pollen period) of anther development in fertile plant of wucai (*B. rapa*); MSp, microspore; PG, pollen grain; T, tapetum. **d**, qRT-PCR analysis of several *BrPERK* genes in the five periods of anther development from wucai fertile plant

The RNA-Seq data from different tissues of *B. rapa* showed that the *BrPERK* members exhibited variable expression patterns; several genes were broadly expressed in all different tissues, while some members were exhibited a more tissue-specific pattern and were especially highly expressed in flower tissue (Fig. 5a). The qRT-PCR results from different organs confirmed the RNA-Seq data, and several *BrPERK* genes were highly expressed in sepals and petals (Fig. 5b). Furthermore, *BrPERK* gene expression differed in different anther developmental stages, with some of these genes exhibiting high expression in the uninucleate, binucleate and mature pollen periods (Fig. 5c-d and S8). Together, these results suggested that some *BrPERK* genes may play important roles in flower development, especially in pollen development.

### Comparative transcriptome analysis of *BrPERK* genes in *B. rapa* male-sterile lines

**Fig. 6:**
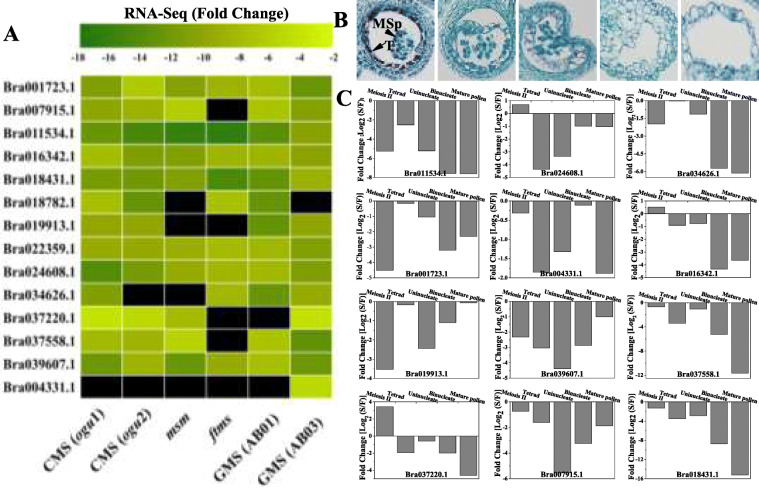
RNA-seq and qRT-PCR analyses of *BrPERK* genes in several male sterility lines of *B. rapa*. **a**, Heat map analysis of *BrPERK* genes in the six different male sterility lines, all the DEGs of *BrPERK* genes were down-regulated; seven (Bra001723.1, Bra011534.1, Bra016342.1, Bra018431.1, Bra022359.1, Bra024608.1 and Bra039607.1) of 14 *BrPERK* DEGs were downregulated in all male sterility plants; the color bar on the top represents fold change values*.***b***.* Transverse sections of anther development in five periods (meiosis II period, tetrad period, uninucleate period, binucleate period and mature pollen period) from Wucai *ogu*-CMS plant; MSp, microspore; T, tapetum. **c**, qRT-PCR analysis of down-regulated *BrPERK* genes in different anther development phases of *B. rapa ogu*-CMS plants (Wucai)

Transcriptome datasets from six different male-sterile lines were analyzed, including two *ogura* cytoplasmic male-sterile (*ogu-*CMS) lines (wucai and turnip), two genic male-sterile (GMS) lines (AB01 and AB03) and two male-sterile mutants (*msm* and *ftms*). A total of 14 differentially expressed genes (DEGs) of *BrPERK*s were detected, and seven of them were downregulated in all male-sterile (MS) plants (Fig. 6 and S9A). qRT-PCR analysis of different developing buds showed that these *BrPERK* genes presented variable expression patterns, and most of them were downregulated after the tetrad period (Fig. 6b and c). These results demonstrated that these downregulated *BrPERK* genes participated in anther development.

### Regulatory subnetworks involving *BrPERK*s and other genes

**Fig. 7:**
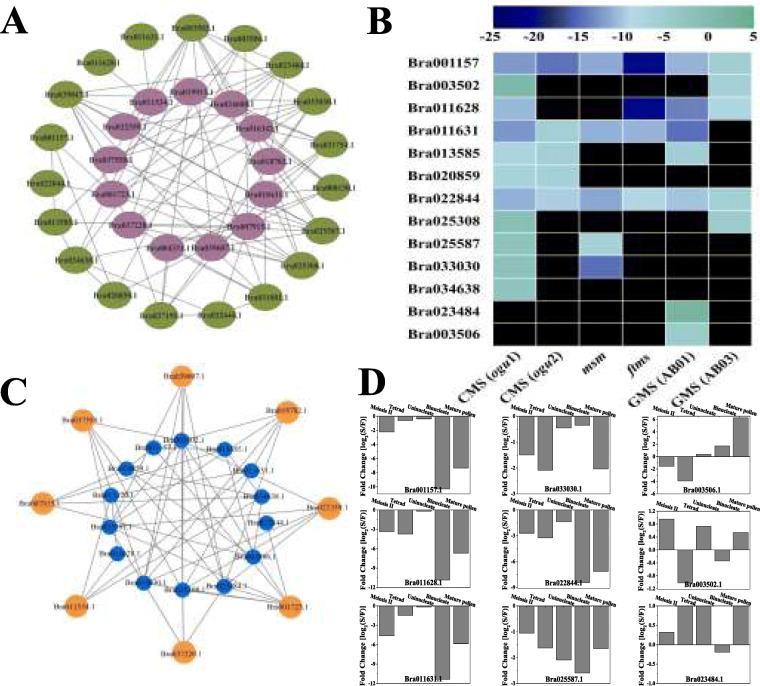
Co-expression, RNA-seq data and qRT-PCR analyses of *BrPERK* co-expressed genes. **a**. Co-expression network of *BrPERK* co-expressed genes was visualized; Nodes are connected by lines to show the interaction of that gene to other co-expressed gene; Reddish brown color indicates 13 down-regulated *BrPERK* genes and green color represent their co-expressed genes. **b**, Heat map analysis of *BrPERK* co-expressed genes in the six different male sterility lines; the color bar on the top represents fold change values*.***c**. Co-expression network of *BrPERK* co-expressed genes; Orange color indicates 8 down-regulated *BrPERK* genes and blue color represent their co-expressed genes. **d**, qRT-PCR analysis of down-regulated *BrPERK* co-expressed genes in *B. rapa ogu*-CMS plants (Wucai)

To gain further insight into the roles of these downregulated *BrPERK* genes in anther development, interaction networks of the *BrPERK* genes in Chinese cabbage were constructed. From the STRING database, 19 coexpressed genes were identified (Fig. 7a). RNA-Seq data from 6 male-sterile lines and qRT-PCR results were analyzed to assess the expression patterns of these genes. Ten genes were identified in at least 2 male lines (Fig. 7b), and most of the genes were downregulated in these male sterile buds (Fig. 7b). The interaction network of these genes with *BrPERK*s and their expression levels during pollen development are shown in Fig. 7c and d, respectively. Gene Ontology (GO) analysis indicated that 8 genes were enriched in pectinesterase activity, nucleic acid binding, cation binding, the membrane, and protein kinase activity; Kyoto Encyclopedia of Genes and Genomes (KEGG) analysis showed that three genes were involved in starch and sucrose metabolism, metabolic pathways, and pentose and glucuronate interconversion (Table S6).

## Discussion

In the present study, we successfully identified 25 *BrPERK* members from the whole-genome sequence of *B. rapa* and analyzed their biophysical properties (Table S3). However, 6 more *BrPERK* members were identified in our analyses that have been reported in previous studies [1]. Among the 25 *BrPERK*s, 6 members (Bra022359, Bra037558, Bra034626, Bra011374, Bra004022 and Bra037291) were shorter, which generally lacked partial sequences in the 5′ flanking region (Fig. 3c); all of these proteins are extracellular proteins (Table S3) and exhibit similar patterns of the main motif distribution (Fig. 3b). These shortened proteins might exist because partial sequences were deleted in the process of gene duplication [33, 34]. Nevertheless, these 6 genes still belong to the *PERK* gene family, and they were retained in the subsequent analyses. Unlike *AtPERK* members [1, 13, 16], all of the proline-rich regions of BrPERKs contained less than 2 Ser-Pro_(2–3)_ motifs in the 5′ flanking region (data not shown).

In addition to *B. rapa* and *A. thaliana*, we identified *PERK*s in 14 other Brassicaceae species (Table S1), and the number of *PERK*s did not differ greatly in these species except in three polyploid plant species, suggesting that the *PERK* family is well conserved in these species [17]. The phylogenetic tree of a total of 418 PERKs showed that Group III among the four subgroups is an ancient clade, similar to the findings from a previous phylogenetic tree of 207 PERKs from 15 other plant species [15]. Interestingly, in Group III, most of the Brassicaceae species presented only one *PERK* member, except for *C. rubella* (n = 8) and *T. salsuginea* (n = 7) and three polyploid plant species, *B. juncea* (n = 18), *B. napus* (n = 19) and *C. sativa* (n = 20) (Fig. 1, Table S2), which exhibited far more *PERK* members than other diploid plant species (Fig. S1, Table S1). These results revealed that the *PERK* gene family is affected by polyploidy and has experienced segmental and genome duplication in these species [15, 35].

The phylogenetic tree of the three *Brassica* species with *A. thaliana* showed that Groups 1–3 included almost equal numbers of *PERK*s in these three *Brassica* species, while Group 4 lacked an *AtPERK* gene (Fig. 2); the *PERK* genes of this group might acquire new functions via divergence following duplication [33, 34, 36, 37]. Furthermore, the sequence logos of the conserved PERK amino acid residues showed that the *PERK* genes are evolutionarily conserved among the *Brassica* species and *A. thaliana* (Fig. S2). This situation can be attributed to the fact that *B. rapa* underwent whole-genome duplication twice in the Brassicaceae lineage and experienced an additional whole-genome triplication event [32, 34].

Additionally, we estimated the divergence times of the orthologous *PERK* pairs between *B. rapa* and *A. thaliana*, *B. nigra*, and *B. oleracea* (Fig. 4 and S7), which showed *Ka*/*Ks* ratios less than 0.35 and indicating the occurrence of strong purifying selection (Fig. S6, Table S5), reflecting the strong control exerted over these genes in evolution [38]. Moreover, the average divergence times of *B. rapa* from *A. thaliana*, *B. nigra*, and *B. oleracea* were estimated to be 17.12, 11.96 and 8.95 Mya, respectively (Table S5, Fig. S6), indicating that *Brassica* species shared a common ancestor and exhibit different differentiation times [32, 35, 39, 40]. It has been reported that the divergence time of the genomes of *B. rapa* and *A. thaliana* was approximately 13 to 17 Mya [41]. These results imply that the *PERK* gene family might be a good candidate molecular reference for analysis of plant evolution. In addition, we analyzed *BrPERK* gene duplication events within the *B. rapa* genome, which are thought to play important roles in the amplification of the genome [42, 43]. In *B. rapa*, 13 paralog pairs of duplicated *BrPERK* genes have been retained, but none of the gene pairs exhibit tandem duplication (Fig. S5A), suggesting that segmental duplication events have played a leading role in *BrPERK* family evolution, similar to what is observed in the IQD gene family of *B. rapa* [44].

It has been reported that *AtPERK* members are involved in several biochemical pathways related to developmental processes in *A. thaliana*, such as pathways that are active in root, rosette leaf, stem and pollen tissues [1, 11, 16]. In *B. rapa*, the functions of *BrPERK*s were explored on the basis of RNA-Seq datasets and qRT-PCR results (Fig. 5). Among different tissues, the gene expression levels of different *BrPERK* genes clearly differed, and some of these genes were very highly and specifically expressed in reproductive organs. These results were similar to those obtained in *A. thaliana* [11, 16] and *Gossypium hirsutum* [15], suggesting that some *PERK* genes play important roles in reproductive developmental processes and might be related to sterility and fertility.

Thus, we assayed 6 RNA-Seq datasets from *B. rapa* male-sterile mutants [26, 28], GMS [29, 30] and CMS [27, 31] lines, and found that 9 *BrPERK* genes were downregulated in at least five MS lines (Fig. 6a). Among these genes, Bra024608.1 and Bra016342.1 were paralogous genes and they were all orthologs of *IGI1* (AT1G23540); Bra022359.1, Bra001723.1 and Bra037558.1 were also paralogous genes, and they were all orthologs of *AtPERK6* (Fig. 4, Table S5). *IGI1* is strongly expressed in the anthers, and the *igi1* mutant exhibited semisterility and smaller siliques [22]. In addition, a phosphoproteomic study of mature *Arabidopsis* pollen grains showed that *AtPERK6* (AT3G18810) was phosphorylated during pollen grain development [23]. In our study, Bra024608.1 and Bra016342.1 were found to be downregulated during anther development from the tetrad period onward, and Bra001723.1 and Bra037558.1 were downregulated beginning in the meiosis II period (Bra022359.1 could not be detected; Fig. 6b and c). Thus, these results suggested that the four *BrPERK* genes might be related to male sterility.

Further analysis of the coexpression network revealed 19 genes that were coexpressed with 13 downregulated *BrPERK*s (except Bra004331.1 in Fig. 6a) screened by STRING (Fig. 7a), and 13 coexpressed genes were identified in the 6 male-sterile RNA-Seq datasets, almost all of which were also downregulated (Fig. 7b). Among these coexpressed genes, no gene coexpressed with Bra024608.1 and Bra016342.1 was screened; nevertheless, 7 genes coexpressed with Bra001723.1 and Bra037558.1 were identified (Fig. 7c, Table S6). In addition, it was reported that Bra001723.1 (Unigen37636) was participated in anther and pollen development [27], and this gene was involved in starch and sucrose metabolism (ko00500) and other metabolic pathways (ko01100) (Table S6). These results demonstrated that Bra001723.1 and Bra037558.1 interact in the regulation of anther and pollen development and the downregulation of these genes might be related to male sterility in *B. rapa*. However, further research is required to investigate the detailed regulatory mechanisms of Bra001723.1 and Bra037558.1 related to male sterility in *B. rapa*.

## Conclusions

In this study, we identified 418 *PERK* gene family members in 16 Brassicaceae species and characterized 25 *BrPERK* genes in *B. rapa*. Analyses of phylogenetic relationships, conserved motifs, gene structures, *cis*-elements, gene duplication and synteny were performed to comprehensively evaluate their biophysical properties in *B. rapa*. Additionally, expression pattern assays based on RNA-Seq and qRT-PCR data revealed that some *PERK*s present tissue-specific patterns and are involved in reproductive development, especially pollen development. Combined analyses of RNA-Seq datasets from six male-sterile lines of *B. rapa* demonstrated that 13 *BrPERK* genes were downregulated in the buds of at least four male-sterile lines, possibly indicating crucial roles in pollen tissue. The expression profiles of these *BrPERK* genes showed that Bra024608.1, Bra016342.1, Bra001723.1 and Bra037558.1 were downregulated during anther development, suggesting that their abnormal expression might be related to male sterility. Furthermore, the coexpression networks assay exhibited that only Bra001723.1 and Bra037558.1 can be detected the coexpressed genes among these four *PERK* genes. These results provided basic information for studying the mechanism of Bra001723.1 and Bra037558.1 function in the male sterility of *B. rapa*.

## Methods

### Identification of *PERK* genes in 16 Brassicaceae species

The sequences of *AtPERK* family proteins were downloaded from the TAIR website following Nakhamchik et al. [16] and served as queries in BLAST searches to identify *PERK* members in the other 15 Brassicaceae species in the *Brassica* Database (BRAD) [32]. After the removal of redundant sequences, all candidate protein sequences were verified using the SwissProt database [45] and the SMART [46], Pfam [47] and InterProScan tools [48]. The confirmed protein sequences were aligned and used to generate a phylogenetic tree in MEGA 7.0 [49]. The conserved amino acid sequence logos were constructed with the WEBLOG tool [50].

### Structural analysis of PERKs

The biophysical properties, protein localization sites, signal peptides, and transmembrane helices of the proteins were predicted using the ExPASy program [51], ProtComp (v6), PSORT Prediction, SignalP [52] and TMHMM Servers (v2.0), respectively. Information on the exon/intron structure, conserved motifs and domains of BrPERKs was obtained from BRAD with the MEME [53] and CDD [54] tools. The predicted motifs were annotated by InterProScan [48]. Tbtools (v0.6679) [55] was used for visual analysis. The promoter regions (1.5 kb upstream sequences) of all *BrPERK* genes were used to predict *cis*-acting elements in the PlantCARE database [56], and promoter structures were obtained with the GSDS (v2.0) [57] tool.

### Genomic distribution and collinearity analyses

The distribution of the *BrPERK* genes on chromosomes was searched and plotted using Tbtools. Synteny analysis among the three *Brassica* crops and *A. thaliana* genomes was conducted via all˗against˗all BLASTP comparisons. The entire protein sequences were used as queries to search the corresponding protein sequence data (*E* < 1e^− 5^, top 5 matches). Collinear pairs were extracted using MCScanX [58] to identify syntenic blocks and duplications within the PERKs.

### Calculation of *K*_*a*_*/K*_*s*_ values

The rates of synonymous (*K*_*s*_) and nonsynonymous (*K*_*a*_) substitutions were calculated for duplicated *BrPERK* genes with the *KaKs* calculator [59]. *K*_*s*_ values > 2.0 were discarded to avoid the risk of substitution saturation, and the divergence time was calculated according to Khan et al. [35] and Yuan et al. [44].

### RNA-Seq data analysis

Seven raw RNA-Seq datasets of *B. rapa* plant were obtained from the NCBI Gene Expression Omnibus (GEO) and Sequence Read Archive (SRA) [26–31, 60]. One of these datasets consisted of the genome-wide transcriptomes of tissues [60], and the other six datasets consisted of the transcriptomes of male-sterile lines, including two male-sterile mutants [26, 28], two GMS lines [29, 30] and two *ogu*-CMS lines [27, 31]. These male-sterile lines are no pollen phenotype and their anther abortion occurs during the tetrad period. The raw data were filtered and mapped onto the *B. rapa* genomes (V1.5 and V3.0). Gene expression levels were estimated using Reads Per Kilobase of exon model per Million mapped reads (FPKM) values [61], and differentially expressed genes (DEGs) were determined with the DESeq or DEGseq software package [62]. The transcriptome expression of *PERK*s was illustrated via heatmap analysis [27].

### Microscopy observations, gene expression and interaction network analyses

The *ogu*-CMS line 4-2B and its 4-2A maintainer line from wucai (*B. rapa* ssp. *Chinensis* var. *rosularis* Tsen) were used as the plant materials in this study, as described previously [63, 64]. Different developmental stages of buds from CMS and fertile lines were used for paraffin section and qRT-PCR analyses (Table S7) according to our previous reports [27, 63, 65]. The interaction network associated with *BrPERK* genes was constructed using the STRING database [66] and Cytoscape software [67].

## Supplementary information

**Additional file 1: Table S1.** Gene locus ID of 16 Brassicaceae plants from *Brassica* Database.

**Additional file 2: Table S2.** Statistics of PERKs in different groups of phylogenetic tree from 16 Brassicaceae species.

**Additional file 3: Table S3.** Biophysical properties of *BrPERK* genes.

**Additional file 4: Table S4.** Characterization of the promoter *cis*-element prediction of *BrPERK* genes.

**Additional file 5: Table S5.** Orthologous / paralogous in *B. rapa* and *A. thaliana*, *B. nigra* and *B. oleracea*.

**Additional file 6: Table S6.** GO and KEGG enrichment analyses of *BrPERK*s co-expressed genes.

**Additional file 7: Table S7.** List of all primers used in this study.

**Additional file 8: Figure S1.** Number and percentage of PERK proteins across the 16 Brassicaceae species.

**Additional file 9: Figure S2.** Sequence logos of conserved amino acid residues in *Arabidopsis thaliana* and three *Brassica* species (*B. rapa*, *B. nigra* and *B. oleracea*).

**Additional file 10: Figure S3.** Sequence logos of conserved amino acid residues in BrPERKs.

**Additional file 11: Figure S4.***Cis*-acting regulatory element analysis of *BrPERK*s promoters by PlantCARE.

**Additional file 12: Figure S5.** Chromosomal location (A) and gene duplication (B) of *BrPERK* genes on their corresponding chromosomes.

**Additional file 13: Figure S6.***Ka*/*Ks* values and divergence times of orthologous / paralogous gene pairs. A, Br-Br; B, Br-At; C, Br-Bni; D, Br-Bo.

**Additional file 14: Figure S7.***BrPERK* gene duplication analysis between *B. rapa* (AA) and *A. thaliana*, *B. nigro* (BB) and *B. oleracea* (CC).

**Additional file 15: Figure S8.** qRT-PCR analysis of three *BrPERK* genes in the five periods of anther development.

**Additional file 16: Figure: S9.** Venn plots of *BrPERK* DEGs (A) and *BrPERK* co-expression genes (B) in six *B. rapa* male sterile lines.

## Data Availability

All data generated or analyzed during this study are included in this published article [and its supplementary information files].

## Abbreviations

BRAD: *Brassica* Database
CMS: Cytoplasmic male sterile
DEG: Differentially expressed gene
FPKM: Reads per kilobase of exon model per million mapped reads
GEO: Gene Expression Omnibus
GMS: Genetic male sterile
GO: Gene ontology
KEGG: Kyoto encyclopedia of genes and genomes
LRR-RK: Leucine-rich repeat receptor kinase
ML: Maximum likelihood
MW: Molecular weight
MS: Male-sterile
Mya: Million years ago
PERK: Proline-rich extension-like receptor protein kinase
pI: Isoelectric point
qRT-PCR: Quantitative real-time PCR
RK: Receptor-like kinase
RNA-Seq: RNA sequencing
SRA: Sequence read archive

## References

[1] Borassi C, Sede AR, Mecchia MA, Salgado Salter JD, Marzol E, Muschietti JP, Estevez JM (2016). An update on cell surface proteins containing extensin-motifs. J Exp Bot.

[2] Hohmann U, Lau K, Hothorn M (2017). The structural basis of ligand perception and signal activation by receptor kinases. Annu Rev Plant Biol.

[3] Osakabe Y, Yamaguchishinozaki K, Shinozaki K, Tran LSP (2013). Sensing the environment: key roles of membrane-localized kinases in plant perception and response to abiotic stress. J Exp Bot.

[4] Muschietti JP, Wengier DL (2018). How many receptor-like kinases are required to operate a pollen tube. Curr Opin Plant Biol.

[5] Gish LA, Clark SE (2011). The RLK/Pelle family of kinases. Plant J.

[6] Shiu SH, Bleecker AB (2001). Plant receptor-like kinase gene family: diversity, function, and signaling. Science's Stke.

[7] Shin Han S, Bleecker AB (2003). Expansion of the receptor-like kinase/Pelle gene family and receptor-like proteins in *Arabidopsis*. Plant Physiol.

[8] Smakowska-Luzan E, Mott GA, Parys K, Stegmann M, Howton TC, Layeghifard M, Neuhold J, Lehner A, Kong J, Grunwald K (2018). An extracellular network of *Arabidopsis* leucine-rich repeat receptor kinases. Nature..

[9] Brandt B, Hothorn M (2016). SERK co-receptor kinases. Curr Biol.

[10] Peng X, Wang M, Li Y, Yan W, Chang Z, Chen Z, Xu C, Yang C, Wang Deng X, Wu J, et al. Lectin receptor kinase OsLecRK-S.7 is required for pollen development and male fertility. J Integr Plant Biol. 2019. 10.1111/jipb.12897. 10.1111/jipb.12897 31833176

[11] Humphrey TV, Haasen KE, Aldeabrydges MG, Sun H, Zayed Y, Indriolo E, Goring DR (2015). PERK-KIPK-KCBP signalling negatively regulates root growth in *Arabidopsis thaliana*. J Exp Bot.

[12] Humphrey TV, Bonetta DT, Goring DR (2007). Sentinels at the wall: cell wall receptors and sensors. New Phytol.

[13] Silva NF, Goring DR (2002). The proline-rich, extensin-like receptor kinase-1 (PERK1) gene is rapidly induced by wounding. Plant Mol Biol.

[14] Haffani YZ, Silva-Gagliardi NF, Sewter SK, Aldea MG, Zhao Z, Nakhamchik A, Cameron RK, Goring DR (2006). Altered expression of PERK receptor kinases in *Arabidopsis* leads to changes in growth and floral organ formation. Plant Signal Behav.

[15] Qanmber G, Liu J, Yu D, Liu Z, Lu L, Mo H, Ma S, Wang Z, Yang Z (2019). Genome-wide identification and characterization of the *PERK* gene family in *Gossypium hirsutum* reveals gene duplication and functional divergence. Int J Mol Sci.

[16] Nakhamchik A, Zhao Z, Provart NJ, Shiu S-H, Keatley SK, Cameron RK, Goring DR (2004). A comprehensive expression analysis of the *Arabidopsis* Proline-rich Extensin-like receptor kinase gene family using bioinformatic and experimental approaches. Plant Cell Physiol.

[17] Florentino LH, Santos AA, Fontenelle MR, Pinheiro GL, Zerbini FM, Baracat-Pereira MC, Fontes EP (2006). A PERK-like receptor kinase interacts with the geminivirus nuclear shuttle protein and potentiates viral infection. J Virol.

[18] Ling B, Guozeng Z, Yun Z, Zhaopei Z, Wei W, Yanyan D, Zhongyi W, Chun-Peng S (2009). Plasma membrane-associated proline-rich extensin-like receptor kinase 4, a novel regulator of Ca^2+^ signalling, is required for abscisic acid responses in *Arabidopsis thaliana*. Plant J.

[19] Bai L, Zhou Y, Song C (2009). *Arabidopsis* proline-rich extensin-like receptor kinase 4 modulates the early event toward abscisic acid response in root tip growth. Plant Signal Behav.

[20] Qin Y, Leydon Alexander R, Manziello A, Pandey R, Mount D, Denic S, Vasic B, Johnso MA, Ravishanka P (2009). Penetration of the stigma and style elicits a novel transcriptome in pollen tubes, pointing to genes critical for growth in a pistil. PLoS Genet.

[21] Won S, Lee Y, Lee H, Heo Y, Cho M, Cho H (2009). Cis-element- and transcriptome-based screening of root hair-specific genes and their functional characterization in *Arabidopsis*. Plant Physiol.

[22] Indeok H, Soo Young K, Cheol Soo K, Yoonkyung P, Giri Raj T, Seong-Ki K, Hyeonsook C (2010). Over-expression of the *IGI1* leading to altered shoot-branching development related to MAX pathway in *Arabidopsis*. Plant Mol Biol.

[23] Mayank P, Grossman J, Wuest S, Boisson‐Dernier A, Roschitzki B, Nanni P, Nühse T, Grossniklaus U (2012). Characterization of the phosphoproteome of mature *Arabidopsis* pollen. Plant J.

[24] Chen L, Liu YG (2014). Male sterility and fertility restoration in crops. Annu Rev Plant Biol.

[25] Wu Y, Min L, Wu Z, Yang L, Zhu L, Yang X, Yuan D, Guo X, Zhang X (2015). Defective pollen wall contributes to male sterility in the male sterile line 1355A of cotton. Sci Rep.

[26] Tan C, Liu Z, Huang S, Li C, Ren J, Tang X, Liu W, Peng S, Feng H (2018). Pectin methylesterase inhibitor (PMEI) family can be related to male sterility in Chinese cabbage (*Brassica rapa* ssp. *pekinensis*). Mol Gen Genomics.

[27] Chen G, Ye X, Zhang S, Yuan L, Zhu S, Hou J, Wang C (2018). Comparative transcriptome analysis between fertile and CMS flower buds in Wucai (*Brassica campestris* L.). BMC Genomics.

[28] Huang S, Peng S, Liu Z, Li C, Tan C, Yao R, Li D, Li X, Hou L, Feng H. Investigation of the genes associated with a male sterility mutant (*msm*) in Chinese cabbage (*Brassica campestris ssp. pekinensis*) using RNA-Seq. Mol Genet Genomics. 2019. 10.1007/s00438-019-01618-z. 10.1007/s00438-019-01618-z 31673754

[29] Liu C, Liu Z, Li C, Zhang Y, Feng H (2016). Comparative transcriptome analysis of fertile and sterile buds from a genetically male sterile line of Chinese cabbage. In Vitro Cell Dev Biol Plant.

[30] Zhou X, Liu Z, Ji R, Feng H (2017). Comparative transcript profiling of fertile and sterile flower buds from multiple-allele-inherited male sterility in Chinese cabbage (*Brassica campestris* L. ssp. *pekinensis*). Mol Gen Genomics.

[31] Lin S, Miao Y, Su S, Xu J, Jin L, Sun D, Peng R, Huang L, Cao J (2019). Comprehensive analysis of Ogura cytoplasmic male sterility-related genes in turnip (*Brassica rapa ssp. rapifera*) using RNA sequencing analysis and bioinformatics. Plos One.

[32] Wang XW, Wang HZ, Wang J, Sun RF, Wu J, Liu SY, Bai YQ, Mun JH, Bancroft I, Cheng F (2011). The genome of the mesopolyploid crop species *Brassica rapa*. Nat Genet.

[33] Khan N, Hu C-M, Khan WA, Wang W, Ke H, Dong H, Zhang Z, Hou X (2018). Genome-wide identification, classification, and expression pattern of homeobox gene family in *Brassica rapa* under various stresses. Sci Rep.

[34] Cheng F, Mandáková T, Wu J, Xie Q, Lysak MA, Wang X (2013). Deciphering the diploid ancestral genome of the mesohexaploid *Brassica rapa*. Plant Cell.

[35] Khan N, Ke H, Hu C-M, Naseri E, Haider MS, Ayaz A, Khan WA, Wang J, Hou X. Genome-wide identification, evolution, and transcriptional profiling of *PP2C* gene family in *Brassica rapa*. Biomed Res Int. 2019:2965035. 10.1155/2019/2965035 PMC6470454 31073524

[36] Conant G, Wolfe K (2008). Turning a hobby into a job: how duplicated genes find new functions. Nat Rev Genet.

[37] Yang Z, Qian G, Qin W, Yang Z, Yuan C, Lu L, Ge X, Zhang C, Wu Z, Li F (2017). Genome-wide analysis of *WOX* genes in upland cotton and their expression pattern under different stresses. BMC Plant Biol.

[38] Liu T, Yu H, Xiong X, Yue X, Yu Y, Huang L, Cao J (2018). Genome-wide identification, molecular evolution, and expression profiling analysis of pectin methylesterase inhibitor genes in *Brassica campestris ssp. chinensis*. Int J Mol Sci.

[39] Beilstein MA, Nagalingum NS, Clements MD, Manchester SR, Sarah M (2010). Dated molecular phylogenies indicate a Miocene origin for *Arabidopsis thaliana*. PNAS..

[40] Yang YW, Lai KN, Tai PY, Li WH (1999). Rates of nucleotide substitution in angiosperm mitochondrial DNA sequences and dates of divergence between *Brassica* and other *Angiosperm* lineages. J Mol Evol.

[41] Town CD, Cheung F, Maiti R, Crabtree J, Haas BJ, Wortman JR, Hine EE, Althoff R, Arbogast TS, Tallon LJ (2006). Comparative genomics of *Brassica oleracea* and *Arabidopsis thaliana* reveal gene loss, fragmentation, and dispersal after polyploidy. Plant Cell.

[42] Zhao T, Wang J, Zhang B, Hou X (2018). Genome-wide analysis of lectin receptor-like kinases in tomato (*Solanum lycopersicum*) and its association with the infection of tomato yellow leaf curl virus. Plant Mol Biol Rep.

[43] Mühlhausen S, Kollmar M (2013). Whole genome duplication events in plant evolution reconstructed and predicted using myosin motor proteins. BMC Evol Biol.

[44] Yuan J, Liu T, Yu Z, Li Y, Ren H, Hou X, Li Y (2019). Genome-wide analysis of the Chinese cabbage IQD gene family and the response of BrIQD5 in drought resistance. Plant Mol Biol.

[45] Bairoch A, Apweiler R (2000). The SWISS-PROT protein sequence database and its supplement TrEMBL in 2000. Nucleic Acids Res.

[46] Letunic I, Doerks T, Bork P (2015). SMART: recent updates, new developments and status in 2015. Nucleic Acids Res.

[47] Finn RD, Coggill P, Eberhardt RY, Eddy SR, Mistry J, Mitchell AL, Potter SC, Punta M, Qureshi M, Sangradorvegas A (2016). The Pfam protein families database: towards a more sustainable future. Nucleic Acids Res.

[48] Jones P, Binns D, Chang H, Fraser M, Li W, McAnulla C, McWilliam H, Maslen J, Mitchell A, Nuka G (2014). InterProScan 5: genome-scale protein function classification. Bioinf..

[49] Kumar S, Stecher G, Tamura K (2016). MEGA7: molecular evolutionary genetics analysis version 7.0 for bigger datasets. Mol Bio Evol.

[50] Crooks GE, Hon G, Chandonia JM, Brenner SE (2019). WebLogo: A sequence logo generator. Genome Res.

[51] Gasteiger E, Hoogland C, Gattiker A, Duvaud S, Wilkins MR, Appel RD, Bairoch A. Protein Identification and Analysis Tools on the ExPASyServer. In: Walker J.M. (eds) The Proteomics Protocols Handbook. Springer Protocols Handbooks. 2005: Humana Press.

[52] Armenteros JJA, Tsirigos KD, Sønderby CK, Petersen TN (2019). Ole Winther SB, Heijne Gv, Nielsen H. SignalP 5.0 improves signal peptide predictions using deep neural networks. Nat Biotechnol.

[53] Bailey TL, Nadya W, Chris M, Li WW (2006). MEME: discovering and analyzing DNA and protein sequence motifs. Nucleic Acids Res.

[54] Marchlerbauer A, Bo Y, Han L, He J, Lanczycki CJ, Lu S, Chitsaz F, Derbyshire MK, Geer RC, Gonzales NR (2017). CDD/SPARCLE: functional classification of proteins via subfamily domain architectures. Nucleic Acids Res.

[55] Chen C, Xia R, Chen H, He Y. TBtools, a Toolkit for Biologists integrating various HTS-data handling tools with a user-friendly interface. bioRxiv. 2018. 10.1101/289660.

[56] Magali L, Patrice D, Gert T, Kathleen M, Yves M, Yves VDP, Pierre R, Stephane R (2002). PlantCARE, a database of plant cis-acting regulatory elements and a portal to tools for in *silico* analysis of promoter sequences. Nucleic Acids Res.

[57] Hu B, Jin J, Guo AY, Zhang H, Luo J, Gao G (2014). GSDS 2.0: an upgraded gene feature visualization server. Bioinf..

[58] Wang Y, Tang H, Debarry JD, Tan X, Li J, Wang X, Lee T, Jin H, Marler BS, Guo H (2012). MCScanX: a toolkit for detection and evolutionary analysis of gene synteny and collinearity. Nucleic Acids Res.

[59] Wang D, Zhang Y, Zhang Z, Zhu J, Yu J (2010). KaKs_Calculator 2.0: a toolkit incorporating gamma-series methods and sliding window strategies. Genomics, Proteom Bioinf.

[60] Tong C, Wang X, Yu J, Jian W, Li W, Huang J, Dong C, Wei H, Liu S (2013). Comprehensive analysis of RNA-seq data reveals the complexity of the transcriptome in *Brassica rapa*. BMC Genomics.

[61] Trapnell C, Williams B, Pertea G, Mortazavi A, Kwan G, van Baren M, Salzberg S, Wold B, Pachter L (2010). Transcript assembly and quantification by RNA-Seq reveals unannotated transcripts and isoform switching during cell differentiation. Nat Biotechnol.

[62] Anders S, Huber W (2010). Differential expression analysis for sequence count data. Genome Biol.

[63] Zhang S, Wang J, Chen G, Ye X, Zhang L, Zhu S, Yuan L, Hou J, Wang C (2019). Functional analysis of a MYB transcription factor *BrTDF1* in the tapetum development of Wucai (*Brassica rapa ssp.)*. Sci Horic.

[64] Chen G, Zeng F, Wang J, Ye X, Zhu S, Yuan L, Hou J, Wang C (2019). Transgenic Wucai (*Brassica campestris* L.) produced via *Agrobacterium*-mediated anther transformation in planta. Plant Cell Rep.

[65] Chen G, Ye X, Zeng F, Wang J, Yuan L, Zhu S, Hou J, Cheng Y, Wang C (2019). Characterization and utilization of a cytoplasmic male sterility line of Wucai (*Brassica campestris* L.). Hortic Environ Biotech.

[66] Szklarczyk D, Gable AL, Lyon D, Junge A, Wyder S, Huerta-Cepas J, Simonovic M, Doncheva NT, Morris JH, Bork P (2018). STRING v11: protein–protein association networks with increased coverage, supporting functional discovery in genome-wide experimental datasets. Nucleic Acids Res.

[67] Shannon P, Markiel A, Ozier O, Baliga NS, Wang JT, Ramage D, Amin N, Schwikowski B, Ideker T (2003). Cytoscape: a software environment for integrated models of biomolecular interaction networks. Genome Res.

