# Supplementary material for: Genome-wide analysis of proline-rich extension-like receptor protein kinase (PERK) in Brassica rapa and its association with the pollen development
Source: BMC Genomics. 2020 Jun 15;21:401. doi: 10.1186/s12864-020-06802-9 (PMC7296749; doi:10.1186/s12864-020-06802-9)
Supplement: Supplementary file 8 — Additional file 8: Figure S1. Number and percentage of PERK proteins across the 16 Brassicaceae species. [file 12864_2020_6802_MOESM8_ESM.pdf]

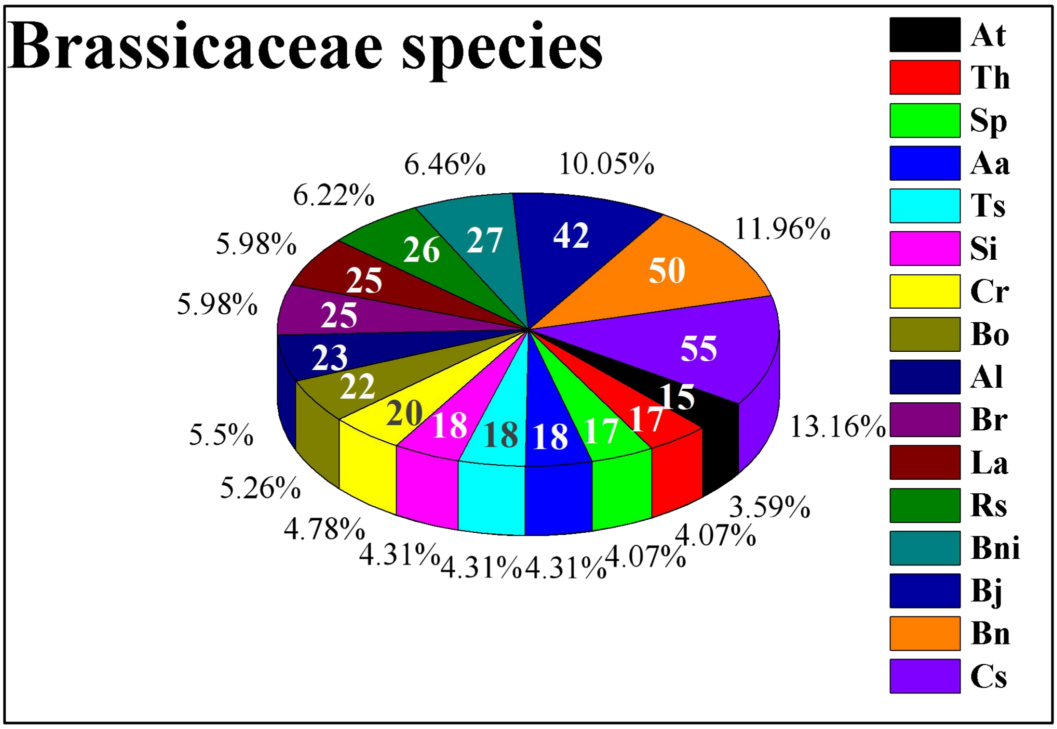

Fig. S1. Number and percentage of PERK proteins across the 16 Brassicaceae species. At, *Arabidopsis thaliana*; Aa, *Aethionema arabicum*; Al, *Arabidopsis lyrata*; Br, *Brassica rapa*; Bni, *Brassica nigra*; Bo, *Brassica oleracea*; Bn, *Brassica napus*; Bj, *Brassica juncea*; Cs, *Camelina sativa*; Cr, *Capsella rubella*; La, *Leavenworthia alabamica*; Rs, *Raphanus sativus*; Sp, *Schrenkiella parvula*; Si, *Sisymbrium irio*; Th, *Thellungiella halophila*; Ts, *Thellungiella salsuginea*.
