# Supplementary material for: Genome-wide analysis of proline-rich extension-like receptor protein kinase (PERK) in Brassica rapa and its association with the pollen development
Source: BMC Genomics. 2020 Jun 15;21:401. doi: 10.1186/s12864-020-06802-9 (PMC7296749; doi:10.1186/s12864-020-06802-9)
Supplement: Supplementary file 9 — Additional file 9: Figure S2. Sequence logos of conserved amino acid residues in Arabidopsis thaliana and three Brassica species (B. rapa, B. nigra and B. oleracea). [file 12864_2020_6802_MOESM9_ESM.pdf]

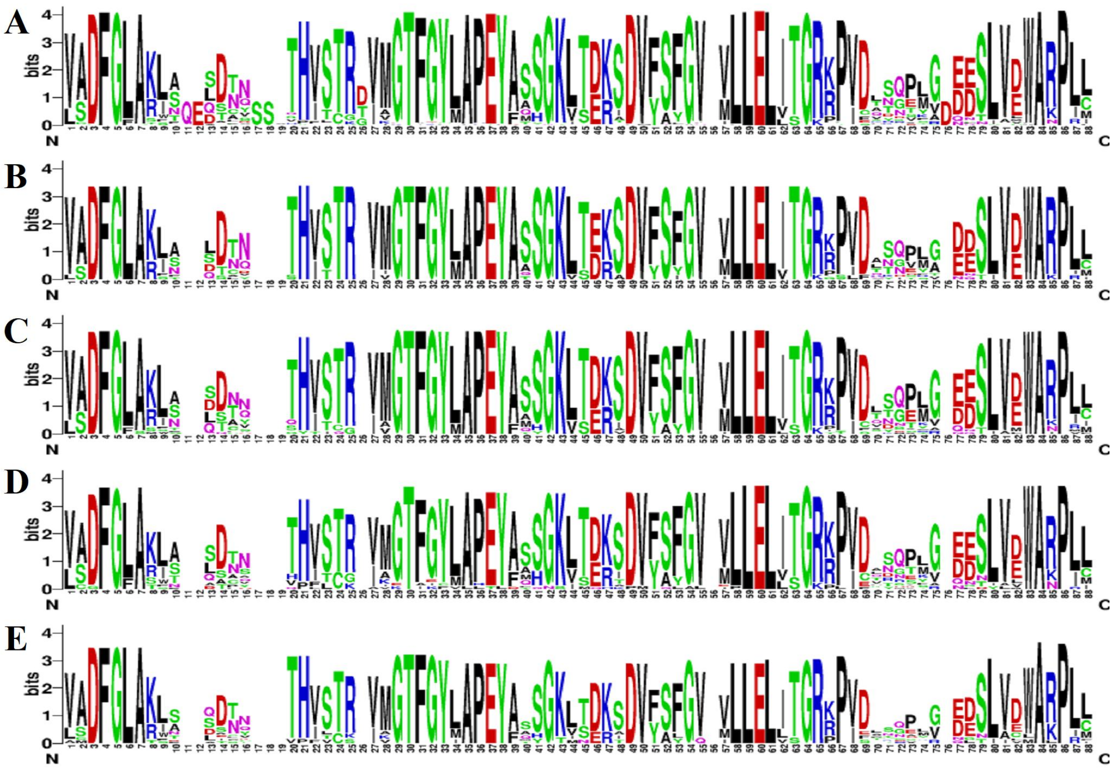

Fig. S2. Sequence logos of conserved amino acid residues in *Arabidopsis thaliana* and three *Brassica* species (*B. rapa*, *B. nigra* and *B. oleracea*). A, four species of *A. thaliana*, *B. rapa*, *B. nigra* and *B. oleracea*. B, *A. thaliana*. C, *B. rapa*. D, *B. nigra*. E, *B. oleracea.*
