# Supplementary figures and images for: Genome-wide analysis of proline-rich extension-like receptor protein kinase (PERK) in Brassica rapa and its association with the pollen development
Source: BMC Genomics. 2020 Jun 15;21:401. doi: 10.1186/s12864-020-06802-9 (PMC7296749; doi:10.1186/s12864-020-06802-9)

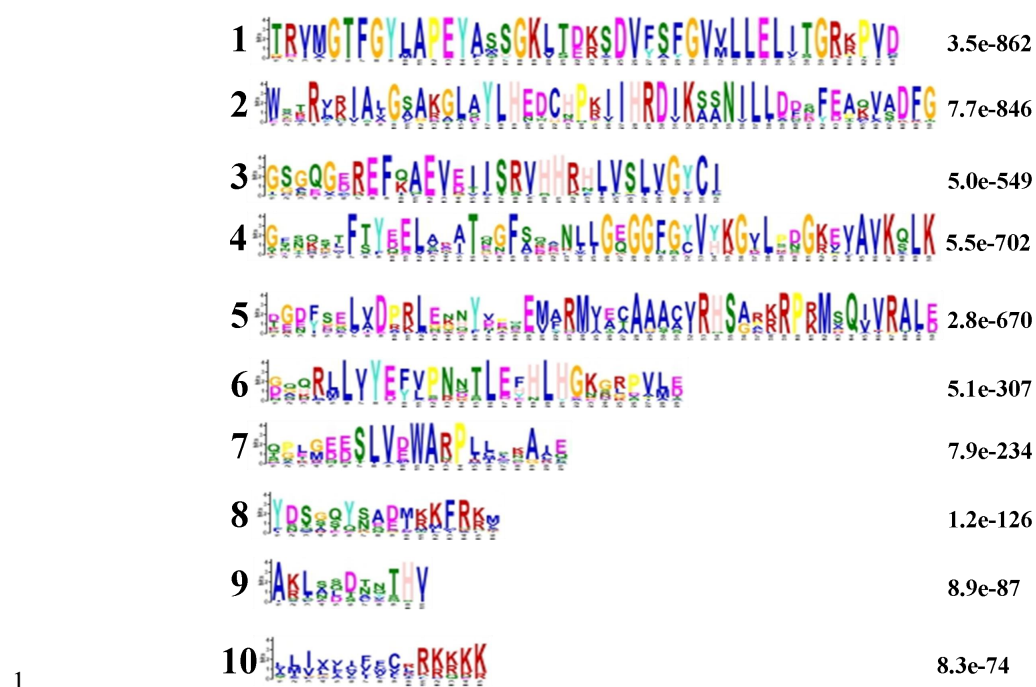

**Fig. S3.** Sequence logos of conserved amino acid residues in BrPERKs.

Supplement: Supplementary file 10 — Additional file 10: Figure S3. Sequence logos of conserved amino acid residues in BrPERKs. [file 12864_2020_6802_MOESM10_ESM.pdf]

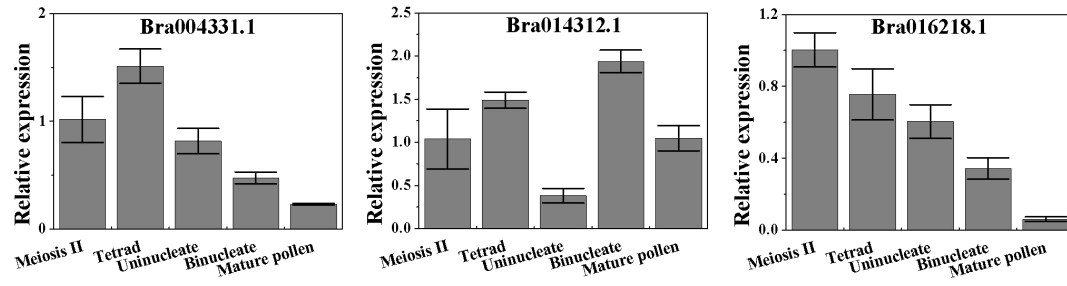

**Fig. S8.** qRT-PCR analysis of three *BrPERK* genes in the five periods of anther development.

Supplement: Supplementary file 15 — Additional file 15: Figure S8. qRT-PCR analysis of three BrPERK genes in the five periods of anther development. [file 12864_2020_6802_MOESM15_ESM.pdf]
