# Supplementary material for: Genome-wide analysis of proline-rich extension-like receptor protein kinase (PERK) in Brassica rapa and its association with the pollen development
Source: BMC Genomics. 2020 Jun 15;21:401. doi: 10.1186/s12864-020-06802-9 (PMC7296749; doi:10.1186/s12864-020-06802-9)
Supplement: Supplementary file 11 — Additional file 11: Figure S4.Cis-acting regulatory element analysis of BrPERKs promoters by PlantCARE. [file 12864_2020_6802_MOESM11_ESM.pdf]

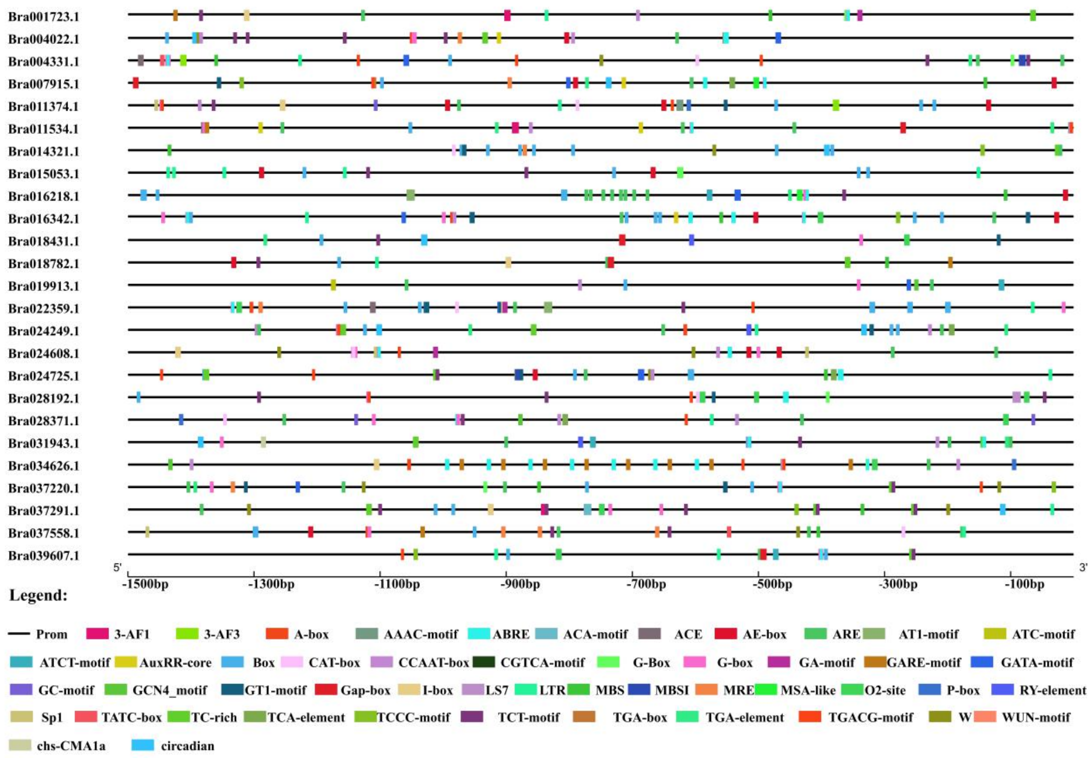

Fig. S4. c*is*-acting regulatory element analysis of *BrPERK*s promoters by PlantCARE. Different *cis*-acting regulatory elements are indicated by different coloured boxes. All the *cis*-acting regulatory elements were calculated and their function were annotated in Table S4.
