# Supplementary material for: Genome-wide analysis of proline-rich extension-like receptor protein kinase (PERK) in Brassica rapa and its association with the pollen development
Source: BMC Genomics. 2020 Jun 15;21:401. doi: 10.1186/s12864-020-06802-9 (PMC7296749; doi:10.1186/s12864-020-06802-9)
Supplement: Supplementary file 12 — Additional file 12: Figure S5. Chromosomal location (A) and gene duplication (B) of BrPERK genes on their corresponding chromosomes. [file 12864_2020_6802_MOESM12_ESM.pdf]

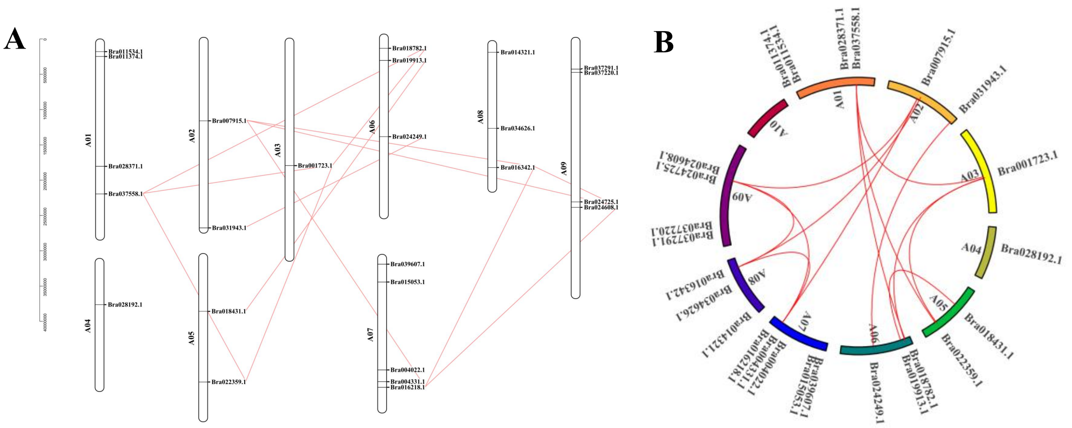

Fig. S5. Chromosomal location (A) and gene duplication (B) of *BrPERK* genes on their corresponding chromosomes.
