# Supplementary material for: Genome-wide analysis of proline-rich extension-like receptor protein kinase (PERK) in Brassica rapa and its association with the pollen development
Source: BMC Genomics. 2020 Jun 15;21:401. doi: 10.1186/s12864-020-06802-9 (PMC7296749; doi:10.1186/s12864-020-06802-9)
Supplement: Supplementary file 13 — Additional file 13: Figure S6.Ka/Ks values and divergence times of orthologous / paralogous gene pairs. A, Br-Br; B, Br-At; C, Br-Bni; D, Br-Bo. [file 12864_2020_6802_MOESM13_ESM.pdf]

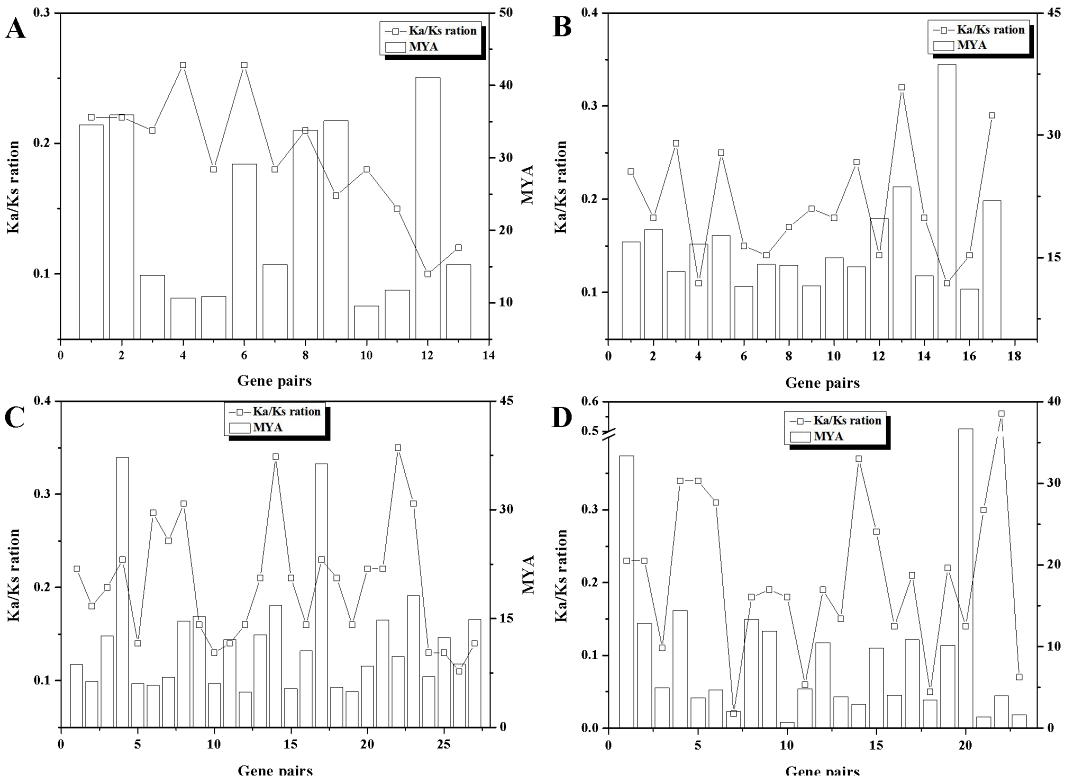


**Fig. S6.** *Ka*/*Ks* values and divergence times of orthologous / paralogous gene pairs. A, Br-Br; B, Br-At; C, Br-Bni; D, Br-Bo.
