# Supplementary material for: Genome-wide analysis of proline-rich extension-like receptor protein kinase (PERK) in Brassica rapa and its association with the pollen development
Source: BMC Genomics. 2020 Jun 15;21:401. doi: 10.1186/s12864-020-06802-9 (PMC7296749; doi:10.1186/s12864-020-06802-9)
Supplement: Supplementary file 14 — Additional file 14: Figure S7.BrPERK gene duplication analysis between B. rapa (AA) and A. thaliana, B. nigro (BB) and B. oleracea (CC). [file 12864_2020_6802_MOESM14_ESM.pdf]

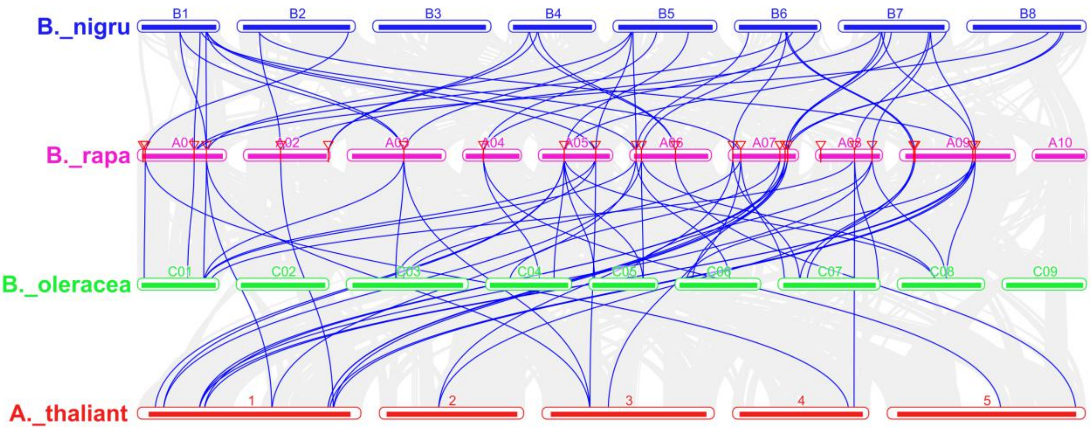

Fig. S7. *BrPERK* gene duplication analysis between *B. rapa* (AA) and *A. thaliana*, *B. nigro* (BB) and *B. oleracea* (CC).
