# Supplementary material for: Genome-wide analysis of proline-rich extension-like receptor protein kinase (PERK) in Brassica rapa and its association with the pollen development
Source: BMC Genomics. 2020 Jun 15;21:401. doi: 10.1186/s12864-020-06802-9 (PMC7296749; doi:10.1186/s12864-020-06802-9)
Supplement: Supplementary file 16 — Additional file 16: Figure: S9. Venn plots of BrPERK DEGs (A) and BrPERK co-expression genes (B) in six B. rapa male sterile lines. [file 12864_2020_6802_MOESM16_ESM.pdf]

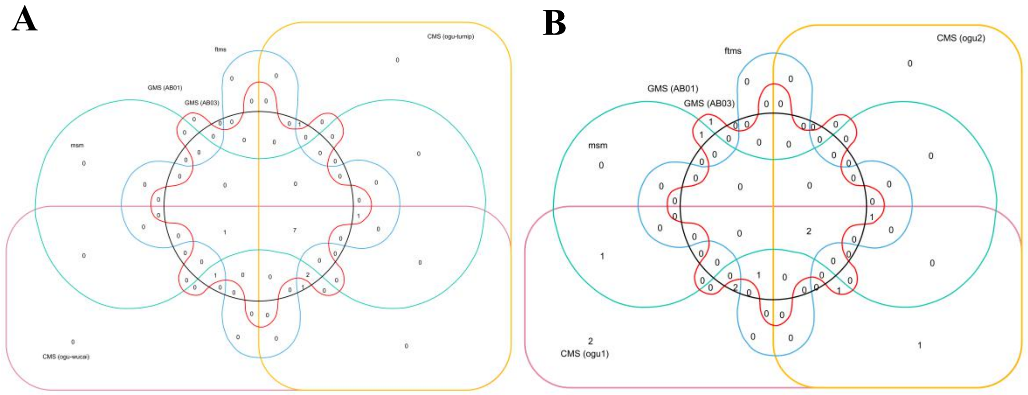

Fig. S9. Venn plots of *BrPERK* DEGs (A) and *BrPERK* co-expression genes (B) in six *B. rapa* male sterile lines.
